# The Emerging Role of Liquid Biopsies in Revolutionising Cancer Diagnosis and Therapy

**DOI:** 10.7759/cureus.43650

**Published:** 2023-08-17

**Authors:** Tejas Shegekar, Sahitya Vodithala, Anup Juganavar

**Affiliations:** 1 Department of Medicine, Jawaharlal Nehru Medical College, Datta Meghe Institute of Higher Education and Research, Wardha, IND; 2 Department of Pathology and Laboratory Medicine, Jawaharlal Nehru Medical College, Datta Meghe Institute of Higher Education and Research, Wardha, IND

**Keywords:** colon cancer, lung cancer, breast cancer, clinical applications, micrornas, exosomes, circulating tumor cells (ctcs), circulating tumor dna (ctdna), cancer, liquid biopsy

## Abstract

A potential non-invasive technique for identifying and tracking cancer is a liquid biopsy. This review article provides a comprehensive overview of the principles, applications, and challenges associated with liquid biopsies. The circulating tumour DNA (ctDNA), circulating tumour cells (CTCs), exosomes, and microRNAs are just a few of the biomarkers we cover in this article that are discovered in liquid biopsies. The clinical application of liquid biopsies in many stages of cancer management, including early cancer identification, therapy selection and response monitoring, and minimum residual illness, is also investigated. The technical advancements in liquid biopsy techniques, including digital polymerase chain reaction (dPCR) and next-generation sequencing (NGS), have improved the sensitivity and specificity of biomarker identification. Liquid biopsies require assistance with cost-effectiveness, sensitivity, and standardisation despite the potential benefits. We talk about these restrictions and potential solutions. In conclusion, liquid biopsies revolutionise personalised therapies and cancer diagnostics by providing a real-time, non-invasive tool for characterising and monitoring tumours. It will be possible to expand the use of liquid biopsies in clinical practises by having a better understanding of their current state and predicted future developments.

## Introduction and background

A revolutionary technique for detecting and monitoring cancer is known as liquid biopsy. The identification and analysis of biomarkers produced by various biofluids, such as blood, urine, or cerebrospinal fluid, is made possible by the use of liquid biopsies. Traditional tissue biopsies, on the other hand, demand invasive procedures. This non-invasive technique offers several benefits, including the possibility of early cancer detection, the opportunity to track the efficacy of treatment in real-time, and the capacity to find any minimally recurrent sickness [1]. The significance of non-invasive cancer screening and detection cannot be overstated. It requires less intrusive procedures, makes cancer diagnostics more patient-friendly and accessible, and makes routine monitoring of the disease's progression and the effectiveness of therapies possible. Liquid biopsies have the potential to change clinical practises by offering vital insights into the heterogeneity, evolution, and genetics of cancer that will assist to direct the development of individualised treatment plans [2]. Numerous biomarkers, each with unique benefits and uses, are included in liquid biopsies, including circulating tumour DNA (ctDNA), circulating tumour cells (CTCs), exosomes, and microRNAs [3]. the likelihood of metastasis and the efficacy of treatment is possible using CTCs, which are complete cancer cells that have detached from the parent tumour [4]. Exosomes are tiny vesicles generated by cancer cells that have a wide range of molecular cargo, including nucleic acids and proteins, and can provide information about the microenvironment around the tumour [5]. Small non-coding RNA molecules also known as microRNAs have a role in gene regulation and can be used as powerful diagnostic and prognostic biomarkers for cancer [6].

In this review article, we will explore the different types of biomarkers utilised in liquid biopsies along with their clinical uses in various cancer types. We will discuss the technological advances in liquid biopsy analysis, such as digital polymerase chain reaction (dPCR) and next-generation sequencing (NGS), and we'll highlight the difficulties and constraints that still need to be solved before they can be widely used [7]. We will also examine the legal and moral issues surrounding liquid biopsies and suggest future research avenues and potential clinical application tactics [8].

## Review

Methodology

**Figure 1:**
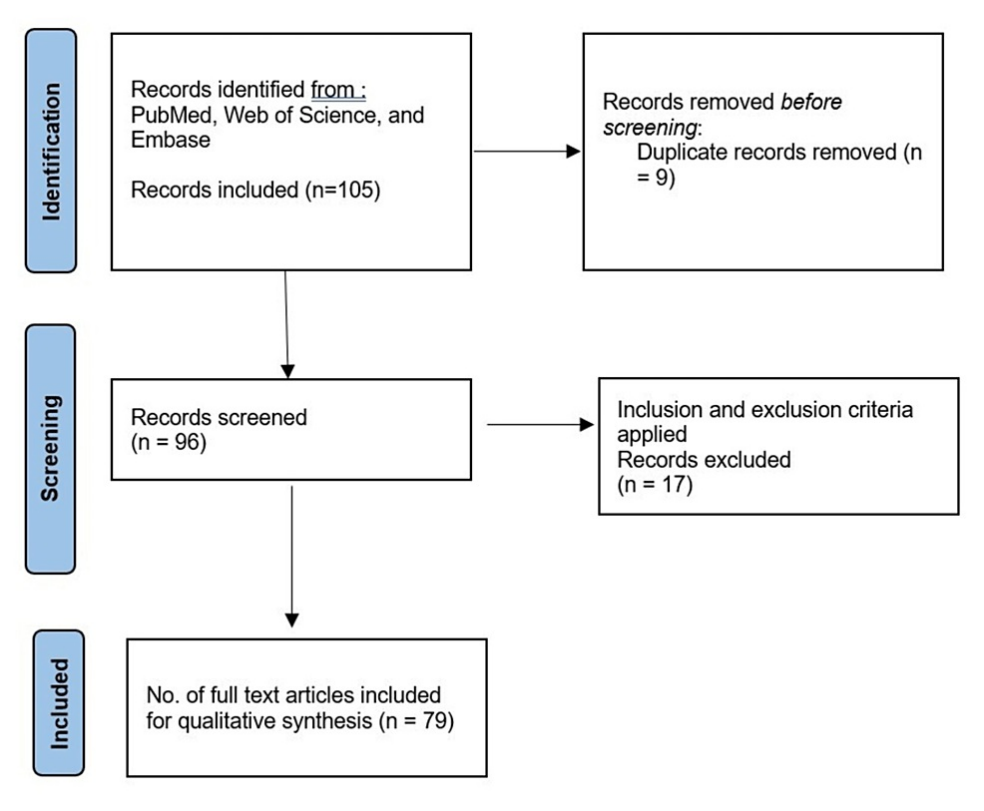
A flowchart of the methodology used for the review

A comprehensive literature search was conducted to identify relevant studies on liquid biopsies for cancer detection and monitoring. The search was performed in major scientific databases, including PubMed, Web of Science, Google Scholar, and Embase. The search was limited to articles published from January 2010 to September 2022 to ensure up-to-date information while capturing the significant developments in the field. The following key terms and MeSH terms were used in various combinations: "liquid biopsy," "circulating tumor DNA," "circulating tumor cells," "exosomes," "microRNAs," "cancer," "clinical applications," and "diagnosis." Boolean operators (AND, OR) were utilised to refine the search and encompass relevant variations of the terms. Inclusion criteria for the studies comprised peer-reviewed articles written in English, focusing on the clinical applications of liquid biopsies in cancer diagnosis and monitoring. Exclusion criteria encompassed studies that were not directly related to liquid biopsy applications, non-human studies, review articles, conference abstracts, and studies without full-text availability. The initial screening of titles and abstracts was carried out to eliminate irrelevant studies. Subsequently, a full-text assessment was conducted to ascertain the studies' eligibility for final inclusion in the review. Following the screening process, a total of 79 articles were included in the final review. The Preferred Reporting Items for Systematic Reviews and Meta-Analyses (PRISMA) flow diagram (Figure 1) below illustrates the study selection process.

Types of biomarkers in liquid biopsies

The detection and analysis of numerous biomarkers originating from tumours found in biofluids are made possible by liquid biopsies. These biomarkers help with cancer diagnosis and surveillance by offering essential insights into the genetic and molecular properties of the tumour. The most common types of biomarkers found in liquid biopsies are the following:

ctDNA

ctDNA represents small fragments of tumour DNA released into the bloodstream by apoptotic or necrotic tumour cells [2]. The examination of ctDNA can reveal information on several facets of cancer treatment. It has demonstrated promise for the early detection of cancer, making it possible to detect tumour-specific changes even at an early stage when tissue samples might not be practical [2]. In addition to monitoring treatment response and identifying minimum residual disease, ctDNA analysis can also be used to determine minimal residual conditions that may go undetected by conventional imaging techniques [9]. For the detection and analysis of ctDNA, numerous techniques have been developed. Identifying particular mutations and genomic changes is made possible by NGS methods such as targeted sequencing, whole-exome sequencing, and whole-genome sequencing [10]. Another effective method for measuring ctDNA is the dPCR, which has a high sensitivity and specificity for identifying uncommon mutant alleles [11].

CTCs

CTCs are intact cells detached from the primary tumour and entered the bloodstream. They stand for a discrete population of cells that can reveal important details about the traits of the tumour, its propensity for metastasis, and its response to therapy. In the area of cancer research and clinical practice, there has been a tremendous increase in interest in the study of CTCs in liquid biopsies. Various approaches, including immunomagnetic separation, microfluidic devices, and filtration procedures, can isolate CTCs from peripheral blood samples. Different molecular and cellular assays can be used to characterise CTCs after they have been identified to learn more about their phenotypic, genetic variations, and functional characteristics. A poor prognosis and an elevated risk of metastasis have been linked to the presence of CTCs in the circulation in several cancer types, including breast, prostate, lung, and colorectal cancer [12,13]. Studies have demonstrated the predictive relevance of CTCs by establishing a correlation between more significant CTC counts and advanced disease stages and worse treatment results [14]. Molecular profiling and individualised treatment plans are other benefits of CTC analysis. CTCs can be examined for genetic changes like mutations, amplifications, and deletions that may help choose which targeted medicines to use. Additionally, examining the gene expression patterns in CTCs can shed light on the biology of the tumour and aid in the identification of possible treatment targets [15].

Additionally, CTCs have been investigated to assess therapy efficacy and identify minimally residual disease (MRD). Early indicators of therapy effectiveness or the formation of treatment resistance can be seen in changes in CTC counts or phenotypic traits during or after treatment. Additionally, the persistence of CTCs after the end of treatment may point to the development of MRD, necessitating additional therapy or more stringent surveillance [16]. Functional analysis of CTCs can also reveal information about their capacity to colonise metastatic sites, persist in circulation, and invade neighbouring tissues. This knowledge is essential for comprehending the mechanisms underlying cancer spread and developing targeted therapies to stop the metastatic process [17].

Exosomes

Multiple body fluids including blood, urine, saliva contain extracellular vesical secreted by tumour cell called exosomes. Cancer diagnosis, prognosis, and treatment monitoring have become easy due to analysis of exosomes that are found in liquid biopsies. Proteins, lipids, nucleic acids, and metabolites comprise the cargo carried by exosomes, reflecting the parent cells' molecular features. They can deliver this cargo to recipient cells, modifying cellular processes and affecting tumour development, invasion, and metastasis [5]. Exosomes from liquid biopsies can be isolated and characterised to acquire tumour-derived biomarkers and learn more about the tumour microenvironment. Exosomes have the potential to serve as cancer detection biomarkers, according to numerous research. In exosomes separated from patient samples, abnormal expression of particular proteins, nucleic acids (such as microRNAs), and cancer-related mutations can be found [18,19]. For example, exosomal microRNAs have shown promise in distinguishing between cancer and healthy individuals and predicting prognosis in various cancer types, including breast, lung, and pancreatic cancer [20,21].

MicroRNAs

Small non-coding RNA molecules called microRNAs (miRNAs) are essential for post-transcriptional gene control. They have been linked to a number of biological functions, including growth, differentiation, and the development of diseases like cancer. MiRNAs are desirable biomarkers for non-invasive cancer diagnosis because they are stable in bodily fluids such as blood, urine, and saliva. Numerous studies have shown specific miRNA signatures linked to various cancer types. For instance, miR-21 has been recommended as a diagnostic and prognostic biomarker since it is often elevated in a number of malignancies, including breast, lung, and colorectal cancer [22,23]. MiRNAs have the potential to aid in cancer biology research in addition to helping in cancer diagnosis. A number of essential pathways connected to tumour development, invasion, and metastasis are regulated by certain miRNAs. For instance, it is well known that members of the miR-200 family are essential for inhibiting metastasis and the epithelial-mesenchymal transition in various cancer types [24].

Furthermore, miRNAs have demonstrated potential as prognostic indicators for therapy response. Differences in miRNA expression patterns can reveal important details about the effectiveness of the treatment and the evolution of drug resistance. For instance, miR-155 has been linked to chemotherapy resistance in several malignancies, including breast and lung cancer [25,26]. Comprehensive miRNA profiling in liquid biopsies is now possible due to the development of high-throughput methods like microarray analysis and next-generation sequencing. These methods make finding miRNA signatures linked to certain cancer types or clinical outcomes easier. There are still challenges in the standardisation of sample collection, RNA extraction, and data analysis methods [27]. Characteristics, advantages, and limitations of biomarkers in liquid biopsies are summarised in Table 1 [1,4,28-33].

**Table 1:** Characteristics, advantages, and limitations of biomarkers in liquid biopsies [1,4,28-33] The table was created by the authors themselves.

Biomarker	Characteristics	Advantages	Limitations
CTCs	Circulating tumor cells	Captures tumor heterogeneity	Rarity of CTCs, challenging isolation
ctDNA	Fragments of tumor DNA	Reflects genetic alterations	Low abundance in early-stage cancers, limited mutation detection
Exosomes	Small extracellular vesicles	Carry tumour-specific molecules	Heterogeneous composition, isolation challenges
microRNAs	Small non-coding RNA molecules	Stable in biofluids, reflects tumor state	Non-specific to tumors, influenced by other conditions

Clinical applications of liquid biopsies

By providing non-invasive ways to gather critical genetic data, liquid biopsies have revolutionised the area of cancer diagnoses and management. The following are some important clinical uses for liquid biopsies:

Early Detection of Cancer

Liquid biopsies provide a way to identify cancer in its earliest stages when it could be easier to cure. To find genetic changes or tumour-specific markers linked to early-stage malignancies, physicians can examine ctDNA and CTCs released into the circulation by tumours. This strategy has shown promise in identifying lung, breast, colorectal, and other malignancies [2,28].

Treatment Selection and Personalised Medicine

By identifying specific genetic mutations, genetic alterations, or biomarkers in tumour cells, liquid biopsies make it possible to pick treatments more precisely. Decisions about targeted therapy, such as choosing certain tyrosine kinase inhibitors for individuals with epidermal growth factor receptor (EGFR) or anaplastic lymphoma kinase (ALK) mutations in lung cancer mutations, might be aided by ctDNA analysis [34].

Monitoring Treatment Response

A dynamic evaluation of therapy response and disease progression is offered by liquid biopsies. To assess the efficacy of treatment, changes in ctDNA levels or particular genetic alternations can be tracked. For instance, a decline in ctDNA levels after therapy commencement implies a positive response, but an increase may indicate resistance or a resurgence of the disease [9].

Detection of MRD

MRD, which is the existence of cancer cells or genetic material after the first therapy, may be found using liquid biopsies. The risk of a disease recurrence or MRD can be assessed using ctDNA or CTC analysis. Decisions on the necessity for additional therapy or surveillance can be guided by this information [7].

Technological advances in liquid biopsy analysis

NGS

As a powerful technique for analysing liquid biopsy samples, NGS enables a thorough and high-throughput study of genetic changes and biomarkers. Here are several NGS-based liquid biopsy technology advancements:

Targeted sequencing panels allow for concurrently investigating several genes or important genomic areas. These panels enrich and capture the desired DNA fragments using specific probes or primers, followed by NGS analysis. This method helps in detecting genetic modifications linked to neoplasms, like mutations, amplifications, fusions, and other alterations. For various cancer types, including lung, breast, and colorectal cancer, targeted sequencing panels have been created, offering a focused and economical method for liquid biopsy analysis [35,36].

Whole exome sequencing (WES) entails sequencing the exome or protein-coding portions of the genome. WES provides for a thorough examination of coding mutations across hundreds of genes by collecting and sequencing the exome. This method makes it possible to detect both well-known and undiscovered genetic changes in liquid biopsy samples. WES has applications in terms of the identification of mutation which can be treated and evaluation of more individualised therapeutic plans [37,38].

Whole genome sequencing (WGS) works on the principle of sequencing the complete genetic makeup and offers a detailed outlook of the genetic environment. Mutations, structural abnormalities, and other genomic rearrangements can be detected with the help of WGS using liquid biopsies samples. WGS is more comprehensive than WES but uses more computing power and resources. Tumours’ clonal development and genetic heterogeneity can be studied through WGS which help in planning more individualised treatment plan [39,40].

Single-cell sequencing technologies have the potential to change the whole perspective of liquid biopsy analysis by making it possible to study the detailed character of individual tumour cells. Individual CTCs or isolated cells from liquid biopsy samples can be sequenced to identify unusual subpopulations, track clonal development, and discover genetic changes. This method offers insight into intratumoral heterogeneity and tumour development [41,42].

NGS may also be used to examine DNA methylation patterns in liquid biopsies, a process known as "methylation profiling." Methylation profiling enables the identification of epigenetic alterations linked to the onset and progression of cancer. Researchers can find aberrant methylation alterations in particular areas or genes, which might be used as biomarkers for the diagnosis and prognosis of cancer [43,44].

Fusion gene detection is a process in which fusion genes originating from chromosomal rearrangements are frequently seen in several forms of cancer. By identifying fusion genes in liquid biopsy samples using NGS-based methods, tumorigenesis-related genomic changes might be better understood. Cancer diagnosis, categorisation, and therapy choice can all be aided by fusion gene identification [45,46].

dPCR

dPCR enables exact and absolute measurement of nucleic acids in a sample [47]. It is a digital substitute for conventional quantitative PCR (qPCR) techniques and has several benefits for analysing liquid biopsy samples. The basic principles of dPCR are the same as those of traditional PCR, except that the PCR reaction is divided into many smaller reactions or droplets, each containing a small number of template molecules [11].

Single-Molecule Sequencing

Single-molecule sequencing methods, such as nanopore sequencing, enable the direct sequencing of individual DNA or RNA molecules without the need for amplification. This technique enables the detection of transcript isoforms, complex genomic rearrangements, and structural alterations in liquid biopsy samples. Real-time sequencing and long-read capacity are advantages [48,49].

Mass Spectrometry-Based Techniques

For analysing liquid biopsies, mass spectrometry imaging (MSI) and liquid chromatography-mass spectrometry (LC-MS) have gained popularity. MSI enables spatial profiling of proteins, peptides, and metabolites in biofluids or tissue samples, providing details on molecular alterations and tumour heterogeneity. LC-MS enables the identification and quantification of proteins, peptides, and small molecules in liquid biopsy samples, opening the door to potential applications in finding biomarkers and monitoring therapeutic response [50].

Techniques for Single-Cell Analysis

Certain cells within a varied population can be identified using single-cell analytic techniques like single-cell proteomics and single-cell RNA sequencing (scRNA-seq). These methods make it possible to characterise intratumoral heterogeneity, identify uncommon cell types, and examine cellular signalling pathways in liquid biopsy samples. Understanding tumour biology, predicting therapy response, and discovering therapeutic targets are all made possible by single-cell research [51,52].

Technological advances in liquid biopsy analysis with key features, advantages, limitations, sensitivity, and specificity are summarised in Table 2 [53-59].

**Table 2:** Technological advances in liquid biopsy analysis NGS: next-generation sequencing, dPCR: digital PCR, DNA: deoxyribonucleic acid, RNA: ribonucleic acid [53-59] The table was created by the authors themselves.

Technology	Key features	Advantages	Limitations	Sensitivity	Specificity
NGS	High-throughput sequencing of DNA/RNA	Detection of various genetic alterations	High-cost, complex data analysis	High	High
dPCR	Partitioning of target molecules	Absolute quantification, high sensitivity	Limited multiplexing capability, lower throughput	High	High
Mass spectrometry	Detection and quantification of proteins/peptides	Multiplex analysis, high sensitivity	Requires target-specific assays, limited coverage	High	High
Antibody-based assays	Detection of specific proteins/antigens	Well-established, broad target coverage	Limited multiplexing, dependence on specific antibodies	Variable	Variable

Biomarker detection and analysis methods

Sample Collection and Processing

The effectiveness of liquid biopsy analysis depends on using the proper sample collecting and processing procedures. Liquid biopsies can be performed using a variety of biofluids, including blood, urine, saliva, and cerebrospinal fluid. Appropriate collection procedures, including standardised protocols for blood draws or urine collection, guarantee the preservation and stability of biomarkers during sample processing. Additionally, it is essential to concentrate and extract biomarkers of interest from the sample using optimised processing procedures, such as centrifugation, filtration, and isolation of certain components (such as plasma, serum, and exosomes) [3,60].

Sensitivity and Specificity Consideration

Accurate and reliable analysis depends on the sensitivity and specificity of biomarker detection techniques. Sensitivity describes a method's capacity to identify low-abundance biomarkers, whereas specificity assesses how well it can distinguish the target biomarker from irrelevant signals. The sensitivity and specificity of various methodologies, including PCR-based approaches, immunohistochemistry, mass spectrometry, and NGS vary depending on the particular biomarkers and their concentration in the sample, as well as the desired clinical application, the suitable detection method must be chosen [61,62].

Data Analysis and Interpretation

Reliable computational techniques and bioinformatics tools are needed to analyse and interpret liquid biopsy data. Large-scale datasets produced by high-throughput methods must be processed, normalised, and analysed to yield helpful information. Data preparation, variant calling, expression profiling, and statistical analysis use bioinformatics pipelines and algorithms. A complete picture of the biomarker landscape may be obtained by integrating several data types, including genomic, epigenomic, transcriptomic, and proteomic data. Furthermore, the discovery of biomarker signatures, prediction models, and clinical decision support systems is facilitated by enhanced data visualisation and machine learning methodologies [63,64].

Clinical utility and evidence in different cancer types

Lung Cancer

Liquid biopsies have demonstrated good therapeutic usefulness in the treatment of lung cancer. They make it possible to identify therapeutically relevant mutations, including those affecting EGFR, ALK, and c-ros oncogene 1 (ROS1) [65]. Additionally, liquid biopsies make it easier to track therapy effectiveness, find sources of resistance, and determine the presence of minimal residual illness [3]. The therapeutic application of liquid biopsy-derived mutations in lung cancer has been supported by several studies that show agreement with tissue biopsy results [66,67].

Breast Cancer

Non-invasive monitoring of breast cancer development and therapy response is provided via liquid biopsies. They can identify gene mutations, including phosphatidylinositol-4,5-bisphosphate 3-kinase catalytic subunit alpha (PIK3CA), human epidermal growth factor receptor 2 (HER2), and breast cancer gene 1/2 (BRCA1/2), providing crucial information for selecting a targeted therapy [68]. CTCs can also be utilised to characterise phenotypes, foretell the possibility of metastatic spread, and determine prognosis [69].

Colorectal Cancer

Liquid biopsies have shown therapeutic value in detecting gene mutations, including Kirsten rat sarcoma viral oncogene homolog (KRAS), neuroblastoma ras viral oncogene homolog (NRAS), and v-raf murine sarcoma viral oncogene homolog B1 (BRAF), which help choose the best-targeted treatment [70]. They also make it possible to measure therapy effectiveness, identify resistance mechanisms, and detect colorectal cancer recurrence early [71]. ctDNA has demonstrated potential as a predictive colorectal cancer biomarker associated with disease stage and survival results [72].

Prostate Cancer

Liquid biopsies have become essential resources in the treatment of prostate cancer. They can identify gene mutations such as transmembrane protease, serine 2, gene-erythroblast transformation-specific-related gene (TMPRSS2-ERG), androgen receptor (AR), and phosphatase and tensin (PTEN), which helps with risk assessment and therapy selection [73]. Additionally, liquid biopsies provide non-invasive therapy response monitoring, MRD detection, and identification of androgen receptor splice variants linked to resistance [74]. Additionally, in advanced prostate cancer, CTC enumeration and characterisation offer prognostic information and direct therapeutic choice [75].

Haematological Cancers

Haematological cancers, such as leukaemia, lymphoma, and multiple myeloma, might benefit significantly from liquid biopsies from a clinical standpoint. They facilitate diagnosis and risk classification by allowing the identification of specific genetic abnormalities, such as gene fusions, mutations, and chromosomal rearrangements. Additionally, liquid biopsies enable the tracking of minimally recurrent illness, treatment efficacy, and the detection of clonal evolution or the establishment of resistance mutations. Additionally, studying ctDNA and CTCs in cases of haematological malignancies offers hope for disease monitoring and relapse prediction [76,77].

The significance of different biomarkers in the clinical outcomes of various cancer are summarised in Table 3 [3,65-77].

**Table 3:** Significance of different biomarkers in clinical outcomes of various cancer MRD: minimal residual disease, ctDNA: circulating tumour DNA, CTCs: circulating tumour cells [3,65-77] The table was created by the authors themselves.

Type of cancer	Biomarkers	Significance	Clinical outcome
Colorectal cancer	ctDNA, CTCs	Detection of KRAS, BRAF mutations, NRAS, and minimal residual disease	Selection of treatment, monitoring of treatment response, assessment of minimal residual disease
Breast cancer	ctDNA, CTCs	HER2 amplification, hormone receptor status, detection of BRCA1/2, PIK3CA	Selection of treatment, monitoring of treatment response, prediction of treatment resistance
Lung cancer	ctDNA, CTCs	EGFR, ALK, and ROS1, treatment response	Early detection, treatment selection, monitoring of treatment response
Prostate cancer	ctDNA, exosomes	Androgen receptor splice variant 7 (AR-V7), TMPRSS2-ERG, AR, PTEN, treatment response	Selection of treatment, monitoring of treatment response, prediction of treatment resistance
Haematological malignancies	ctDNA, CTCs, exosomes, microRNAs	Genetic mutations, MRD	Assessment of treatment response, assessment of disease recurrence, monitoring of minimal residual disease

Challenges and limitations of liquid biopsies

Standardisation of Protocols

The lack of standardised protocols for sample collection, processing, and analysis poses a challenge in liquid biopsies. Variations in pre-analytical and analytical methods can impact the accuracy and reproducibility of results. Standardisation efforts are essential to guarantee consistent and dependable results across various laboratories and clinical contexts [1].

Sensitivity for Low-Frequency Mutations

Liquid biopsies have difficulty identifying low-frequency mutations, especially in tumours in the early stages or those with limited residual illness. Identification and precise quantification of uncommon genetic abnormalities are difficult due to the low levels of ctDNA or CTCs in the blood. Their sensitivity must be improved for liquid biopsy tests to be used more widely in cancer diagnosis and surveillance [78].

Considerations for Cost-Effectiveness

The price of liquid biopsy analysis, which includes sample collection, processing, and specialised laboratory methods, may prevent its widespread use. A few technologies, including NGS, can be expensive and need specialised infrastructure and knowledge. The requirement for recurrent testing and long-term monitoring may further raise total costs [79]. For liquid biopsies to be helpful in everyday clinical practice, a balance between cost-effectiveness and therapeutic usefulness must be struck.

## Conclusions

Liquid biopsies have emerged as a promising approach for non-invasive cancer detection, monitoring, and personalised treatment decision-making. Liquid biopsies provide several advantages over conventional tissue biopsies, including their minimally invasive nature, ability to capture tumour heterogeneity, and potential for longitudinal monitoring through the analysis of ctDNA, CTCs, exosomes, and microRNAs. In this review, we discussed the several kinds of biomarkers used in liquid biopsies including ctDNA, CTCs, exosomes, and microRNAs. Across a variety of cancer types, we investigated their involvement in cancer diagnosis, prognosis, therapy selection, and monitoring. With encouraging outcomes in early identification, treatment response evaluation, and detection of minimal residual illness, liquid biopsies are clinically helpful in the treatment of lung cancer, breast cancer, colorectal cancer, prostate cancer, and haematological malignancies. The area of liquid biopsies does, however, face challenges and limitations. Standardising sample collecting, processing, and analysis procedures are crucial to guarantee accurate and repeatable findings. Enhancing liquid biopsy tests' sensitivity and specificity is still a top objective, especially for low-frequency mutations. The broad implementation of liquid biopsies depends on cost-effectiveness evaluations and incorporation into standard clinical practice.

In conclusion, liquid biopsies have the potential to completely transform the way that cancer is treated by offering non-invasive, continuous, and thorough molecular profiling. They provide new opportunities for early cancer detection, individualised therapy selection, and treatment response tracking. Liquid biopsies are set to become an essential component of conventional clinical practice with additional development, standardisation, and regulatory clearances, eventually enhancing patient outcomes in cancer. The remaining issues should be addressed in future studies, along with confirming the therapeutic value of liquid biopsies in larger patient cohorts and incorporating them into clinical protocols and healthcare systems. Realising the full potential of liquid biopsies and implementing them into better cancer treatment requires collaboration between academics, doctors, regulatory agencies, and industry players.
